# Functionality and Antioxidant Properties of Tilapia (*Oreochromis niloticus*) as Influenced by the Degree of Hydrolysis

**DOI:** 10.3390/ijms11041851

**Published:** 2010-04-26

**Authors:** Mohamed Beva Kelfala Foh, Issoufou Amadou, Betty Mabel Foh, Mohamed Tabita Kamara, Wenshui Xia

**Affiliations:** 1 State Key Laboratory of Food Science and Technology, Jiangnan University, No 1800, Lihu Road, Wuxi, 214122 Jiangsu, China; 2 School of Community Health and Clinical Studies, Njala University, Kowama Campus, Bo, Sierra Leone

**Keywords:** tilapia, fresh minced meat, hot water dip, hydrolysis, antioxidant activity, functional foods

## Abstract

Freeze dried protein powders (Fresh minced meat, FMM and Hot water dip, HWD) from tilapia (*Oreochromis niloticus*) were hydrolyzed by Alcalase 2.4 L (Alc), Flavourzyme (Flav) and Neutrase (Neut), and investigated for antioxidant activity and their functional properties. FMM and HWD hydrolysed by Alc, exhibiting superior antioxidant activity, had estimated degrees of hydrolysis (DH) of 23.40% and 25.43%, respectively. The maximum values of the 1,1-diphenyl-2-picrylhydrazyl (DPPH), 2,2-azino-bis(3-ethylbenzothiazoline-6-sulfonic acid) diammonium salt (ABTS), 3-(2-pyridyl) 5,6-bis(4-phenyl-sulphonic acid)-1,2,4-triazine (ferrozine), radical scavenging activities and metal chelating properties were 86.67%, 91.27% and 82.57%, and 84.67%, 92.60% and 78.00% for FMM and HWD, respectively, with a significant difference (*P* < 0.05) between the samples. Essential amino acids were above the amounts recommended by the Food and Agricultural Organization/World Health Organization (FAO/WHO/UNU) for humans. Lower molecular weight sizes <3,000 Da were more predominant in FMM and HWD hydrolysed by Alc, while in hydrolysed by Flav and Neut they were >8,000 Da. At pH 2, FMM and HWD hydrolysates have varying solubilities above 85% (Alc FMM; 91.33%, Flav FMM; 79.5%, Neut FMM; 83.8% and Alc HWD; 90.45%, Flav HWD; 83.5%, and Neut HWD; 85.8%). They have ‘U’ shaped solubility curves, water holding capacity was in the range of 2.77 and 1.77 mL/g, while oil holding capacity ranged between 3.13 and 2.23 mL/g. FMM and HWD have the highest bulk density of 0.53 and 0.53 for Neutrase and Alcalase 2.4 L, respectively. Foam capacity and stability ranged from 125.5 to 61.4, 138.5 to 45.2, 130.0 to 62.5, and 124.5 to 55.0, 137.5 to 53.3, 129.6 to 62.7 for FMM and HWD hydrolyzed with Alcalase 2.4 L, Flavourzyme and Neutrase, respectively. Tilapia fish protein hydrolysates are thus potential functional food ingredients.

## 1. Introduction

Tilapias inhabit a variety of fresh water habitats. Traditionally they have been of major importance in small scale commercial or subsistence fishing worldwide, especially Africa and Asia. It is the third most widely cultured fish, after carp and salmonids [1]. The global production has been greatly influenced by rapid expansion of Nile tilapia (*Oreochromis niloticus*) and Mossambique tilapia (*Oreochromis mossambicus*), cultured in China, the Phillipines and Egypt [2]. Tilapia fish is nutritious and forms a healthy part of a balanced diet that is high in protein (16–25%), low in fat (0.5–3.0%) and substitutes well in any seafood recipe [3].

Modification of a protein is usually realized by physical, chemical, or enzymatic treatments, which change its structure and consequently its physicochemical and functional properties [4]. Enzymatic hydrolysis has been widely used to improve the functional properties of proteins, such as solubility, emulsification, gelation, water and fat-holding capacities, and foaming ability, and to tailor the functionality of certain proteins to meet specific needs [5]. Series of studies have demonstrated that enzymatic hydrolysis of fish and fish by-products including, capelin [6], salmon protein [7,8], shark protein [9], herring [10], and sardine [11,12] improved their functional properties. The production of Fish Protein Hydrolysates (FPH) under controlled conditions is a way of improving its nutritional value [6].

Apart from fish protein hydrolysates’ functionalities, different sources, such as capelin mackerel and herring have been found to possess antioxidant properties [10,13,14]. Many human diseases are known to be caused by free radicals and the natural antioxidants can act as free radical scavengers. Protein hydrolysates with antioxidant properties, in particular, have become a topic of great interest for the pharmaceutical, health food, as well as the food processing and preservation industries [15,16]. There is also a growing interest in antioxidants from natural sources, which may have less potential health hazard compared with synthetic antioxidants. The bioactive molecules in FPH responsible for antioxidant properties are peptides that are released upon hydrolysis. As a result, the objectives of this study were to investigate the degree of hydrolysis, amino acid content, molecular weight, functional properties, and antioxidant activity of Nile tilapia hydrolysates.

## 2. Results and Discussion

### 2.1. Degree of Hydrolysis (DH)

**Figure 1.**
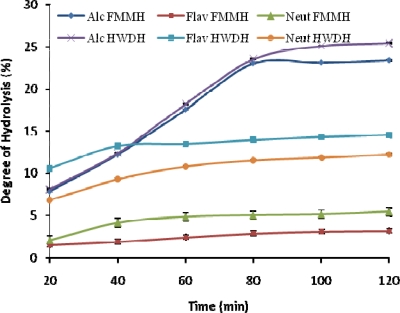
Effect of time on the degree of hydrolysis (DH) of tilapia (*Oreochromis niloticus*) fish protein hydrolysates (TFPH). FMMH: Fresh minced meat hydrolysate; HWDH: Hot water dip hydrolysate Value represent the mean ± standard deviation (SD) of n = 3 duplicate assays (Alc-Alcalase 2.4 L; Flav- Flavourzyme; Neut-Neutrase).

The DH is an important factor highly related with the hydrolysis process yield [6]. The results of the DH are presented in Figure 1. There was an initial rapid increase in DH with increased time relating to the frequency of addition and volume of NaOH used to maintain pH. This indicated that maximum cleavage of peptides occurred within the first hour of hydrolysis. For all proteinases, Alcalase 2.4 L showed the highest value in terms of DH for HWD and FMM (Figure 1). The result agreed with reported enzymatic hydrolyses of fish protein substrates [10,17]. The significant difference (*P* < 0.01) of DH with Alcalase 2.4 L treatment suggested that Alcalase 2.4 L has superior affinity hence, is a more efficient enzyme than Flavourzyme and Neutrase for preparing Nile tilapia protein hydrolysates.

### 2.2. Amino Acid Analysis

The total amino acid composition of Tilapia fish protein hydrolysates (FMM and HWD) are shown in Table 1, along with the recommended essential amino acid composition according to the FAO/WHO/UNU (2007) [18]. It is clear that tilapia fish protein contains all the essential amino acids in good proportion as compared to Sathivel *et al.* [10] with a significant difference (P < 0.05). The results in Table 1 indicate that the amino acid composition of FMM closely resembles to that of HWD. However, glutamic acid, aspartic acid and lysine were found to be abundant, as expected in most fish protein hydrolysates [8,29]. Both FMM and HWD have a well balanced amino acid composition and most of the essential amino acids compositions of their proteins were at a higher level than FAO/WHO/UNU protein and amino acid requirements in human nutrition [18]. The values are generally in accordance with previous studies [6,7,27].

### 2.3. Molecular Weight Distribution

Molecular weight distributions of tilapia fish protein (FMM and HWD) Hydrolysates were determined by SE-HPLC (Table 2) by a TSK gel, 2000SWXL (7.8 × 300 mm) column. The molecular weights for all samples were calculated according to the equation:

(1) $$\text{Log}\;\text{Mol}\;\text{Wt}\;=\;6.70-0.2.14\text{T}\;\text{with}\;{\text{R}}^{2}\;=\;0.9953$$

The rising level of DH corresponds inversely to lower molecular weight distributions. The result in Table 2 shows that hydrolysates from Alcalase 2.4 L had lower molecular weights, below 3,000 Da.

This result also indicated that cleavage of peptide bonds by the proteases had taken place. Hydrolysates from Flavourzyme and Neutrase with low DH (Figure 1) were characterized by a high percentage of peptides with molecular weights ranging from 8,000 Da to 15,000 Da. Different DH and proteases led to different peptide chain lengths which greatly influenced the antioxidant activities of the hydrolysates, corroborating with findings that purified peptides with a molecular weight of less than 1,000 Da from Alaska pollack frame proteins showed the strongest antioxidant activity among the hydrolysate fractions [19].

### 2.4. DPPH Radical Scavenging Activity

A rapid, uncomplicated and inexpensive method to measure antioxidant capacity of food involves the use of the free radical, 2,2-diphenyl-1-picrylhydrazyl (DPPH). DPPH is used to test the ability of compounds to act as free radical scavengers or hydrogen donors, and to evaluate antioxidant activity. DPPH scavenging activity of Tilapia fish protein hydrolysates (FMM and HWD) are listed in Table 3.

The DH data earlier reported (Figure 1) shows a reasonable link to this result. The samples (FMM and HWD) hydrolysed using Alcalase 2.4 L, Neutrase and Flavourzyme show an increasing DPPH radical scavenging activity: 86.67%, 82.00%, 70.20%, and 84.67%, 79.67%, 64.67% for both samples, respectively. There was a significant difference (P < 0.05) between the various samples. Moreover, FMM and HWD hydrolysates produced using Flavourzyme exhibited the lowest DPPH radical scavenging activity. The peptides in tilapia fish protein hydrolysates (FMM, HWD) demonstrated a role as good electron donors and could react with free radicals to terminate the radical chain reaction. DPPH is a stable free radical with a maximum absorbance at 517 nm in ethanol. When DPPH encounters a proton-donating substance, the radical is scavenged and the absorbance is reduced [20]. The results indicated that the tilapia FPH acted as a good electron donor and could react with free radicals to terminate the radical chain reaction, corroborating other findings [21,22].

### 2.5. ABTS Radical Scavenging Activity

Protein hydrolysates from many sources have been found to possess antioxidative activity [21,22]. The ABTS radical assay is a widely used method of screening for antioxidant activity and is reported as a decolorization assay applicable to both lipophilic and hydrophilic compounds [23]. The assay results of the different tilapia FPH after using different enzymes are shown in Table 3. They possesses high ABTS radical scavenging ability (*P* < 0.05) and could reduce more than 80% of the ABTS radicals in the assay media at 66.67 μg/mL sample concentration. The variation between the results of FMM and HWD were significantly different (*P* < 0.05). The findings indicated that 120 min hydrolysis of the samples (FMM and HWD), resulted in reasonable antioxidant ability and moreover, the conditions applied in the hydrolysis are sufficient in making antioxidative FPH from tilapia fish. Similarly, high antioxidative scavenging quality is shown in tilapia fish protein hydrolysates from DPPH and metal chelating activities, exhibiting similar results reported by Klompong *et al*. [24]

### 2.6. Metal-Chelating Activity

A sample concentration of 5 mg/mL was used to determine the metal chelating properties of tilapia fish protein hydrolysates. The results from Table 3 show that tilapia fish protein (FMM and HWD) hydrolysate samples interacted with iron ion. The chelating activity however shows a significant difference (*P* < 0.05) between the samples. Alcalase-treated samples manifested a higher chelating activity (FMM; 82.5%, HWD; 78.0%) than Flavourzyme and Neutrase-treated ones (75.8%, 75.8% and 77.23%, 78.32%) for FMM and HWD respectively. This result also corroborates a linear relationship with the increased DH, lower molecular weight distribution, and high peptide solubility, that is showing higher metal chelating activity [25].

### 2.7. Nitrogen Solubility

**Figure 2.**
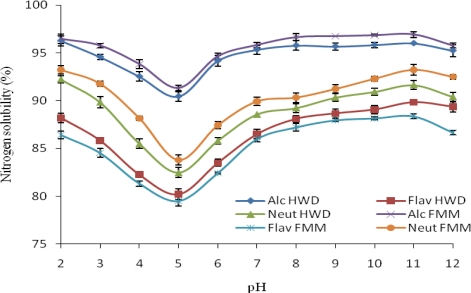
Effect of pH treatment on nitrogen solubility of tilapia fish protein hydrolysates (*Oreochromis niloticus*). Value represent the mean ± standard deviation (SD) of n = 3 duplicate assays. FMMH-Fresh minced meat hydrolysates; HWDH-Hot water dip hydrolysates (Alc-Alcalase 2.4 L; Flav- Flavourzyme; Neut-Neutrase).

An increase in the extent of enzymatic hydrolysis corresponded to a considerable increase in the nitrogen solubility over the pH range studied, indicating a positive relationship (Figure 2). Between pH 4.5 and 5.5 near the isoelectric point (pI) at which the net charge of the original proteins are minimized and consequently, more protein-protein interactions and fewer protein-water interaction occur. FMM and HWD have very similar solubility profiles; exhibiting a “U” shaped curve in which FMM and HWD hydrolyzed with Alcalase 2.4 L have the highest solubility values at alkaline pH. Under acidic conditions, all proteins had solubility above (80%). At pH 6.0, nitrogen solubility increased rapidly with an increase in pH up to 12.0. These trends in solubility are in agreement with [7,9,10]. At pH 11.0, the solubility of FMM reached 96.93%, 93.23%, and 88.33% for Alcalase 2.4 L, Neutrase and Flavoourzyme respectively, whilst solubility for HWD were 96.0%, 91.63% and 89.83% for Alcalase 2.4 L, Neutrase and Flavoourzyme respectively. The maximum solubility was under alkaline conditions. Protein solubility at various pH values may serve as a useful indicator of how well protein hydrolysate will perform when they are incorporated into food systems. The solubility curve is typical of that of most fish protein hydrolysates. Enzymatic protein Hydrolysis leads to smaller peptides, consequently, to more soluble products. This is in accordance with the findings of [6,7] who reported that hydrolysates had an excellent solubility at high degrees of hydrolysis. The pH values influence the charge on the weakly acidic and basic side chain groups [4]. Solubility variations could be attributed to both net charge of peptides, which increase as pH moves away from pI, and surface hydrophobicity, that promotes the aggregation via hydrophobic interaction [26]. Since many functional properties of proteins depend upon their capacity to initially go into solution, the excellent solubility of the FPH suggests that they may have many potential applications in formulated food systems.

### 2.8. Oil/Water Holding Capacity

The ability of protein hydrolysates to absorb oil is an important functionality that influences the taste of the product that is required in various food industries. The OHC for FMM are in the ranges of 2.27, 3.07, 2.77 mL/g for Alcalase 2.4 L, Flavourzyme and Neutrase respectively, with significant difference (*P* < 0.01). HWD hydrolysates have OHC of 2.23, 2.57, 3.13 mL/g for Alcalase 2.4 L, Flavourzyme and Neutrase, respectively (Table 4).

On the other hand, the functional properties of proteins in food systems broadly depend on the water-protein interactions. The ability of protein to imbibe water and retain it against a gravitational force within a protein matrix is known as Water Holding Capacity (WHC). The WHC for FMM is in the range of 2.10, 2.77, 2.57 mL/g for Alcalase 2.4 L, Flavourzyme and Neutrase respectively, with significant difference (*P* < 0.01). HWD hydrolysates have WHC of 1.77, 2.10, 2.57 mL/g for Alcalase 2.4 L, Flavourzyme and Neutrase, respectively, with significant difference (*P* < 0.01).

### 2.9. Emulsifying Capacity (EC)

The ability of proteins to form stable emulsions is important due to the interactions between proteins and lipids in many food systems. An increase in the number of peptide molecules and exposed hydrophobic amino acid residues due to hydrolysis of proteins should contribute to an improvement in the formation of emulsions. From the results, samples hydrolyzed using Alcalase 2.4 L (FMM and HWD) had lower emulsifying capacities of 22.33 and 21.40 mL/0.5g of protein with a significant difference (*P* < 0.01). Samples hydrolyzed with Neutrase and Flavourzyme (FMM and HWD) have an EC of 22.50 and 23.20, and 27.33 and 26.40 mL/0.5g of protein, respectively (Table 4). Our result is in agreement with with [7]. Extensive protein hydrolysis may result in a marked loss of emulsion properties. Though small peptides diffuse to, and absorb fast at the interface, they are less efficient in reducing the interfacial tension due to lack of unfolding and reorientation at the interface that large peptides are [7].

### 2.10. Foam Capacity and Foam Stability (FC/FS)

The formation of protein based foams involves the diffusion of soluble proteins toward the air-water interface and rapid conformational change and rearrangement at the interface; the foam stability requires formation of a thick, cohesive, and viscoelastic film around each gas bubble [27]. Hence, ability to form foam is a function of the configuration of protein molecules. FMM and HWD show a significant difference (*P* < 0.01) in the foaming capacity. Samples hydrolyzed with Alcalase 2.4 L have a FC of 125.5 and 124.5 g/mL, Flavourzyme with FC of 138.5 and 137.5 g/mL and Neutrase 130.2, and 129.6 g/mL for FMM and HWD, respectively. The results imply an increase in surface activity, probably due to the initial greater number of polypeptide chains that arose from partial proteolysis, allowing more air to be incorporated. Similar FC data was obtained in previous studies [27,28].

To have foam stability, protein molecules should form continuous intermolecular polymers enveloping the air bubbles, since intermolecular cohesiveness and elasticity are important to produce stable foams. Foam stability values ranged from 125.5 to 61.4, 138.5 to 45.2, 130.0 to 62.5, and 124.5 to 55.0, 137.5 to 53.3, 129.6 to 62.7 for FMM and HWD hydrolyzed with Alcalase 2.4 L, Flavourzyme and Neutrase, respectively. The FS for tilapia fish protein hydrolysates were within the range of the results reported by Wasswa *et al.* [29].

On the other hand, there was a significant decrease (*P* < 0.05) in the foaming stability, (Alc. FMM, 38.2; Alc. HWD, 37.2 g/mL), (Flav. FMM, 47.3; Flav. HWD, 47.7 g/mL) and (Neut. FMM, 48.0, Neut. HWD, 50.5 g/mL). An opposite effect on the surface activity is probably due to the lower surfactant activity of smaller peptide chains from extensive hydrolysis [28]. These foaming properties suggest that tilapia fish protein hydrolysate is a better foaming agent in protein foods.

### 2.11. Bulk Density

There was a significant difference (*P* < 0.01) among the various samples studied (Table 4). FMM and HWD hydrolyzed with Neutrase and Alcalase 2.4 L shared a comparable and higher bulk density of 0.53 and 0.54 g/mL, while the samples hydrolyzed with Flavourzyme had the lowest (FMM: 0.35 g/mL; HWD: 0.34 g/mL). Furthermore, the bulk density of tilapia fish protein hydrolysates demonstrated lower bulk density compared to tilapia skin protein hydrolysate [29]. Bulk density represents the behavior of a product in dry mixes, and is an important parameter that can determine the packaging requirements of a product. Also it varies with the fineness of particles. High bulk density is unfavorable for the formulation of weaning foods, where low bulk density is required [30].

### 2.12. *In Vitro* Protein Digestibility

*In vitro* protein digestibility of FMMH and HWDH samples were evaluated by the release of TCA-soluble nitrogen, after incubation time of 120 min at 37 °C. Table 4 shows that all protein samples exhibited very good trypsin digestibility. Nonetheless, HWD fractions hydrolysed using Alcalase, Flavourzyme, and Neutrase have digestibility values with trypsin of 93.2%, 89.3%, and 92.83%, whereas FMM fractions showed digestibility values with trypsin of 92.72%, 88.23%, and 92.43%, respectively, with a significant difference (*P* < 0.01). Our results are within the values reported by Aziz *et al*. [31]. The pretreatment undergone by the samples during the cause of hydrolysis improved digestibility of protein and may be attributed to the increase in protein solubility, or structural unfolding of protein molecules [32].

## 3. Experimental

### 3.1. Materials and Methods

Fresh minced meat (FMM) and Hot water dip (HWD) samples were obtained as byproducts from Fresh Nile tilapia, *Oreochromis niloticus*, 480–600 g/fish with length range of 25–30 cm/fish, purchased from a local fresh water product market in Wuxi, China. The fish samples were transported within 24 h after purchased in ice boxes to the School of Food Science and Technology (SFST) laboratory of Jiangnan University, Wuxi, Jiangsu, China. On arrival at the University laboratory, the fresh fish were prepared using the standard handling method; gutted, beheaded, and skin removed before thoroughly washing with clean water to remove contaminants or unwanted particles. Fish muscle was retrieved with care, separating the bones from the meat, chopped into pieces (about 0.25 cm) and divided into two portions. A portion of the chopped meat was dipped in hot water (95 ± 5 °C) and maintained for 15 min (HWD), hence endogenous enzymes were supposedly inactivated and further impurities removed. It was allowed to cool at room temperature, eventually vacuum packed in polyethylene bags, and kept frozen at −20 °C till needed for the experiments. The remaining portion was subjected to mincing using a meat mincer and the pulverized fish meat (homogenate) were also vacuum packed in polyethylene bags (100–250 g per unit), and kept frozen at −20 °C till needed for the experiments. Alcalase 2.4 L from a strain of *Bacillus licheniformis*, Flavourzyme 500 LAPU/g from *Aspergillus oryzae* and Neutrase 1.5 AU/g from *Bacillus subtulis* strain were obtained from Novozymes China Inc. and stored at 4 °C for subsequent analysis. 1,1-Diphenyl-2-picrylhydrazyl (DPPH), 3-(2-pyridyl)-5,6-bis(4-phenylsulphonic acid)-1,2,4-triazine (ferrozine), and 2,2-azino-bis(3-ethylbenzothiazoline-6-sulfonic acid) diammonium salt (ABTS) were obtained from Sigma-Aldrich (Shanghai, China). All chemical reagents used for experiments were of analytical grade.

### 3.2. Preparation of Fish Protein Hydrolysates

HWD and FMM were hydrolyzed with three different enzymes, under the conditions given in Table 5 based on optimum hydrolysis conditions. One hundred grams of tilapia fish were weighed into a vessel immersed in a water bath maintained at an appropriate temperature and 300 mL of distilled water was added to make a suspension. The suspension, for each enzyme applied, was adjusted to a suitable pH and pre heated to an appropriate temperature, then (0.5%, 1%, and 1.5%) enzymes:substrate ratio was added with continuous stirring. Hydrolysis was carried out for 120 min. After hydrolysis, the enzymes were inactivated by placing in boiling water for 15 min. The hydrolysate was allowed to cool down and centrifuged at 7,500 × g for 15 min. at 4 °C with a D-3756 Osterode am Harz model 4515 centrifuge (Sigma, Hamburg, Germany). The tilapia fish protein hydrolysate (FPH) was lyophilized and stored at −20 °C until used. All experiments were performed in triplicate and the results are the average of the three values.

### 3.3. Determination of the Degree of Hydrolysis DH

Reactions were monitored by measuring the extent of proteolytic degradation by means of the DH according to the pH-stat method described by Adler-Nissen [33]. The degree of hydrolysis (*DH%*), is defined as the percent ratio of the number of peptide bonds broken (*h*) to the total number of bonds per unit weight (*h_tot_*), in each case, was calculated from the amount of base consumed [32], as given below:

(2) $$DH(\%)=\frac{V_{b}\times N_{b}}{\alpha \times mP\times h_{\text{tot}}}\times 100$$

where *V*_b_ is base consumption in mL; *N*_b_ is normality of the base; α is average degree of dissociation of the α-NH_2_ groups; *mP* is mass of protein (N × 6.25) in g; and *h*_tot_ is total number of peptide bonds in the protein substrate. All the experiments were performed in triplicate and the results are the average of three values.

### 3.4. Amino Acid Analysis

Amino acid determination commenced by placing samples of FPH (100 mg) for all the samples and 5 mL 6 M HCl in a 50 mL bottle that was sealed. The air was removed by keeping the sample in a vacuum chamber. The sealed samples were placed in an oven at 120 °C for 16 hours to hydrolyze. After hydrolysis, 5 mL of 2 mM norleucine internal standard was added and the solution was filtered in a 0.2 μL Gelman membrane filter. One mL of stock sample was pipetted into a 50 mL borosilicate glass serum bottle and dried in a freeze-drier. One mL of sodium diluent buffer (pH 2.2) was added to the freeze-dried residue and transferred to a 1.5 mL micro-centrifuge tube for HPLC analysis. The prepared samples were injected as 2.5 μL volumes and run on a Waters HPLC (Waters Corporation, Milford, MA, USA) at a flow rate of 0.4 mL/min with a Pickering sodium ion-exchange column of 4 × 150 mm (Pickering Laboratories, Inc., Mountain View, CA, USA) and sodium eluent (pH 3.15 and 7.40). TRIONE^®^ ninhydrin reagent was added with post column instrument (TRIONE^®^ ninhydrin derivatization instrument, Pickering Laboratories, Inc.). The light absorbance of amino acids was detected with an UV Visible detector (Pickering Laboratories Inc.) at 570 nm wavelength and the amino acids were quantified by comparing with standard amino acid profiles. Methionine and cysteine were determined separately by oxidation products according to the performic acid procedure of Moore [34] before hydrolysis in 6 M HCl. Tryptophan was determined after alkaline hydrolysis by isocratic ion-exchange chromatography with *O*-phthalaldehyde derivatization followed by fluorescence detection by Ravindran and Bryden [35]. Amino acid composition was reported as g/100 g of protein.

### 3.5. Determination of Molecular Weight

The samples were determined using a Waters™ 600 E Advanced Protein Purification System (Waters Corporation, Milford, MA, USA). A TSK gel, 2000SWXL (7.8 × 300 mm) column was used with 10% acetonitrile + 0.1% TFA in HPLC grade water as the mobile phase. The calibration curve was obtained by running bovine carbonic anhydrase (29,000 Da), horse heart cytochrome C (12,400 Da), bovine insulin (5800 kDa), bacitracin (1450 Da), Gly-Gly-Tyr-Arg (451 kDa) and Gly-Gly-Gly (189 Da). The total surface area of the chromatograms was integrated and separated into eight ranges (>8,000, 3,000–8,000, 2,000–3,000, 1,000–2,000, 600–1,000, 300–600, 200–300, <200 Da), expressed as a percentage of the total area, Table 2. The results were obtained and processed with the aid of Millennium 32 Version 3.05 software (Vaters Corporation, Milford, MA 01757, USA).

### 3.6. DPPH Radical Scavenging Activity Assay

The method described by Wu *et al.* [14] was used to measure the DPPH radical scavenging activity with a slight modification. FPH samples (FMMH and HWDH) were dissolved in distilled water to obtain a concentration of 40 mg protein/mL. Then 2.0 mL of sample was mixed with 2.0 mL of 0.15 mM DPPH that was dissolved in 95% ethanol. The mixture was then shaken vigorously using a mixer (QT-1 Mixer, Tianchen Technological Co. Ltd. Shanghai, China) and kept in the dark for 25–30 min. The absorbance of the resultant solution was recorded at 517 nm. The scavenging activity was calculated using the following equation:

(3) $$\text{DPPH}\;(\%)=\left(\frac{\text{absorbance}\;\text{of}\;\text{DPPH}-\;\text{absorbance}\;\text{of}\;\text{sample}}{\text{absorbance}\;\text{of}\;\text{DPPH}\;\text{blank}}\right)\times 100$$

where the DPPH blank is the value of 2 mL of 95% ethanol mixed with DPPH solution, the DPPH sample is the value of 2 mL of sample solution mixed with DPPH solution, and the control sample is the value of 2 mL of sample solution mixed with 2 mL of 95% ethanol.

### 3.7. ABTS Radical Scavenging Activity Assay

ABTS radical scavenging activities of FPH samples were determined by the method described Re *et al*. [36], with slight modifications. A stock solution of ABTS radicals was prepared by mixing 5.0 mL of 7 mM ABTS solution with 88 μL of 140 mM potassium persulfate, and keeping in the dark at room temperature for 12–16 h. An aliquot of stock solution was diluted with phosphate buffer (PB, 5 mM, pH 7.4) containing 0.15 M NaCl in order to prepare the working solution of ABTS radicals to an absorbance of 0.70 ± 0.02 at 734 nm. A 65 μL aliquot of tilapia FPH dissolved in the same phosphate buffer (66.67 μg/mL final assay concentration) or only buffer (for the control) was mixed with 910 μL of ABTS radical working solution, incubated for 10 min at room temperature in the dark, and then absorbance was measured at 734 nm. The percent reduction of ABTS^+^ to ABTS was calculated according to the following equation:

(4) $$\text{ABTS}\;(\%)=\left(1-\frac{\text{absorbance}\;\text{of}\;\text{sample}}{\text{absorbance}\;\text{of}\;\text{control}}\right)\times 100$$

### 3.8. Metal-Chelating Activity

The metal-chelating activity of FPH was assessed using the method of Decker and Welch [37]. One mL of peptide solution (5 mg/mL) was first mixed with 3.7 mL of distilled water. Then it was reacted with a solution containing 0.1 mL 2 mM FeCl_2_ and 0.2 mL of 5 mM ferrozine. After 10 min, the absorbance of the reaction mixture was measured at 562 nm. The metal-chelating ability of FPH was calculated as a percentage applying the equation:

(5) $$\text{Metal-chelating}\;\text{ability}\;(\%)=\left(1-\frac{\text{absorance}\;\text{of}\;\text{sample}}{\text{absorbance}\;\text{of}\;\text{control}}\right)\times 100$$

### 3.9. Nitrogen Solubility (NS)

Nitrogen solubility was determined according to the procedure of Diniz and Martin [9], with slight modifications. Samples were dispersed in distilled water (10 g/L) and pH of the mixture was adjusted to 2,3,4,5,6,7,8,9,10,11,12 with either 0.5 N HCL or 0.5 N NaOH while continually shaking (Lab-Line Environ-Shaker; Lab-Line Instrument, Inc., Melrose Park, IL, USA) at room temperature for 35 min. a 25 mL aliquot was then centrifuged at 2,800 g for 35 min. A 15 mL aliquot of the supernatant was analyzed for nitrogen (N) content by the Kjeldahl method and the NS was calculated according to Equation:

(6) $$\text{Nitrogen}\;\text{solubility}\;(\%)=\left(\frac{\text{sup}\;\text{erna}\;\text{tan}t\;(N)\;\text{concentration}}{\text{sample}\;(N)\;\text{concentration}}\right)\times 100$$

### 3.10. Oil-Holding Capacity (OHC)

Oil-holding capacity (OHC) of tilapia FPH was determined as the volume of edible oil held by 0.5 g of material according to the method of Shahidi *et al.* [6]. A 0.5 g sample of each FPH was added to 10 mL soybean oil (Gold Ingots Brand, QS310002012787, Suzhou, P.R. China) in a 50 mL centrifuge tube, and vortexed for 30 s in triplicate. The oil dispersion was centrifuged at 2,800 × g for 25 min. The free oil was decanted and the OHC was determined by weight difference.

### 3.11. Water-Holding Capacity (WHC)

To determine the Water Holding Capacity (WHC) of tilapia FPH, the method outlined by Diniz and Martin [9], was followed with slight modifications. Triplicate samples (0.5 g) of hydrolysates were dissolved with 10 mL of distilled water in centrifuge tubes and vortexed for 30 s. The dispersions were allowed to stand at room temperature for 30 min, centrifuged at 2,800 × g for 25 min. The supernatant was filtered with Whatman No.1 filter paper and the volume retrieved was accurately measured. The difference between initial volumes of distilled water added to the protein sample and the volume retrieved. The results were reported as mL of water absorbed per gram of protein sample.

### 3.12. Emulsifying Capacity (EC)

Emulsifying capacity was measured using the procedure described by Rakesh and Metz [38], with modification. A 0.5 g of each freeze-dried sample was transferred into a 250 mL beaker and dissolved in 50 mL of 0.5 N NaCl, and then 50 mL of soybean oil (Gold Ingots Brand, QS310002012787, Suzhou, P.R. China) was added. The homogenizer equipped with a motorized stirrer driven by a rheostat Ultra-T18 homogenizer (Shanghai, China) was immersed in the mixture, and operated for 120 s at 10,000 rpm to make an emulsion. The mixture was transferred to centrifuge tubes, maintained in water-bath at 90 °C for 10 min and then centrifuged at 2800 × g for 20 min. Emulsifying capacity was calculated as in equation:

(7) $$EC\;=\frac{V_{A}-V_{R}}{W_{S}}$$

where *V_A_* is the volume of oil added to form an emulsion, *V_R_* is the volume of oil released after centrifugation, and *W_S_* is the weight of the sample.

### 3.13. Foaming Capacity (FC) and Foam Stability (FS)

Estimation of foaming capacity was done following the method of Bernardi Don *et al.* [39], with minor modifications. Thirty mL of 30 g/L aqueous dispersion was mixed thoroughly using an Ultra-Turrax 25 homogenizer at 9,500 rpm for 3 min in a 250 mL graduated cylinder. The total volume of the protein dispersion was measured immediately after 30 s. The difference in volume was expressed as the volume of the foam. Foam stability was determined by measuring the fall in volume of the foam after 60 min.

### 3.14. Bulk Density (BD)

Bulk density of freeze-dried tilapia FPH was estimated with approximately 3 g of each sample packed into 25 mL graduated cylinders by gently tapping on the lab bench 10 times. The volume was recorded and bulk density was reported as g/mL of the sample.

### 3.15. *In Vitro* Protein Digestibility (IVPD)

*In vitro* protein digestibility (IVPD) was carried out according to the method described by Elkhalil *et al.* [40], with slight modifications. Twenty mg of tilapia FPH (FMMH and HWDH) samples were digested in triplicate in 10 mL of trypsin (0.2 mg/mL in 100 mM Tris-HCl buffer, pH 7.6). The suspension was incubated at 37 °C for two hours. Hydrolysis was stopped by adding 5 mL 50% trichloroacetic acid (TCA). The mixture was allowed to stand for 30–35 min at 4 °C and was then centrifuged at 10,000 × g for 25 min using a D-3756 Osterode AM Harz Model 4515 Centrifuge (Sigma, Hamburg, Germany). The resultant precipitate was dissolved in 5 mL of NaOH and protein concentrate was measured using the Kjeldahl method. Digestibility was calculated as follows:

(8) $$\text{Protein}\;\text{digestibility}\;(\%)=\frac{\left(A-B\right)}{A}\times 100$$

where *A*: total protein content (mg) in the sample and *B*: total protein content (mg) in the TCA precipitate.

### 3.16. Statistical Analysis

The results obtained were subjected to one-way analysis of variance (ANOVA) using SPSS 18.0 statistical software package (SPSS Inc, Chicago, IL, USA). Each value was determined by at least three replicates. Results were given as mean ± standard deviation.

## 4. Conclusions

The study demonstrated that Alcalase 2.4 L is a suitable protease for use in the production of tilapia (*Oreochromis niloticus*) muscle hydrolysates that exhibit a significant antioxidant activity and due to its its functionality, it can serve as a good source of quality food ingredients and also provide desirable characteristics to food products. Antioxidants block the process of oxidation by neutralizing free radicals. The hydrolysates from FMM and HWD revealed a wide range of molecular weights polypeptides with an appreciable level of solubility, high digestibility, fat absorption, foaming capacity, emulsifying capacity and valuable antioxidant properties that can compete with hydrolysates and protein powders currently available in the market. On the whole, endogenous enzyme inactivation in HWD did not manifest significant differences in antioxidant properties and functionalities.

## Figures and Tables

**Figure 3.**
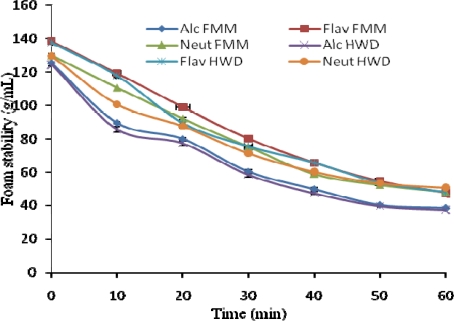
Foam stability of fish protein hydrolysates (*Oreochromis niloticus*). Value represent the mean ± standard deviation (SD) of n = 3 duplicate assays FMMH-Fresh minced meat hydrolysate; HWDH-Hot water dip hydrolysate. Alc-Alcalase 2.4 L; Flav-Flavourzyme; Neut-Neutrase.

**Table 1.** Total amino acid composition of Tilapia Fish protein Hydrolysates (g/100 g Protein).

**Amino Acids**	**Alcalase 2.4 L**		**Flavourzyme**		**Neutrase**		**FAO/WHO/UN^a^**	

	FMM	HWD	FMM	HWD	FMM	HWD	Child	Adult

**Essential Amino acids**								
**Isoleucine**	3.79 ± 0.08^d^	3.48 ± 0.01^c^	3.12 ± 0.02^a^	3.22 ± 0.11^a^^b^	3.29 ± 0.01^b^	3.26 ± 0.01^b^	3.0	3.0
**Leucine**	8.04 ± 0.01^a^^b^	8.23 ± 0.03^b^^c^	8.23 ± 0.03^b^^c^	8.69 ± 0.06^d^	7.88 ± 0.12^a^	8.26 ± 0.14^c^	6.0	5.9
**Lysine**	9.08 ± 0.01^a^	10.14 ± 0.01^c^	10.51 ± 0.03^d^	10.81 ± 0.06^e^	9.87 ± 0.01^b^	10.53 ± 0.01^d^	4.8	4.5
**Methionine**	2.96 ± 0.02^e^	2.66 ± 0.01^c^	2.34 ± 0.01^a^	2.52 ± 0.01^b^	2.73 ± 0.03^d^	2.63 ± 0.01^c^	2.3^b^	1.6^b^
**Met + Cys**	3.60 ± 0.07^d^	3.35 ± 0.06^c^	2.85 ± 0.01^a^	3.11 ± 0.02^b^	3.22 ± 0.01^c^	3.29 ± 0.02^c^		
**Phenylalanine**	3.78 ± 0.02^e^	3.20 ± 0.01^d^	3.13 ± 0.01^c^	2.99 ± 0.01^a^	3.12 ± 0.01^b^^c^	3.09 ± 0.01^b^	4.1^c^	3.8^c^
**Phe + Tyr**	6.70 ± 0.03^d^	5.31 ± 0.02^c^	4.64 ± 0.01^a^	4.66 ± 0.06^a^	5.24 ± 0.02^b^^c^	5.16 ± 0.01^b^		
**Threonine**	4.58 ± 0.09^d^	4.38 ± 0.04^c^	3.95 ± 0.01^a^	4.09 ± 0.02^b^	4.21 ± 0.05^b^	4.39 ± 0.04^c^	2.5	2.3
**Valine**	4.30 ± 0.01^f^	4.10 ± 0.01^e^	3.77 ± 0.01^a^	3.91 ± 0.01^b^	3.94 ± 0.01^c^	3.98 ± 0.01^d^	2.9	3.9
**Histidine**	2.28 ± 0.06^d^	2.09 ± 0.02^a^^b^	2.03 ± 0.03^a^	2.09 ± 0.02^a^^b^	2.17 ± 0.06^b^^c^	2.23 ± 0.01^c^^d^	1.6	1.5
**Tryptophan**	5.42 ± 0.01^e^	2.79 ± 0.02^d^	1.32 ± 0.01^a^	1.67 ± 0.02^b^	2.78 ± 0.02^d^	2.33 ± 0.02^c^	0.66	0.6
**Nonessential Amino Acid**								
**Alanine**	6.44 ± 0.02^a^	6.81 ± 0.02^b^	7.98 ± 0.01^f^	7.61 ± 0.01^e^	7.06 ± 0.03^c^	7.20 ± 0.03^d^		
**Arginine**	5.76 ± 0.02^a^	5.97 ± 0.01^b^	6.08 ± 0.02^c^	6.13 ± 0.04^c^^d^	6.17 ± 0.01^d^	6.17 ± 0.01^d^		
**Aspartic acid^d^**	9.96 ± 0.02^c^	10.25 ± 0.01^d^	9.91 ± 0.01^b^	10.39 ± 0.02^e^	9.85 ± 0.02^a^	10.59 ± 0.02^f^		
**Cysteine^e^**	0.66 ± 0.03^c^	0.55 ± 0.00^b^	0.51 ± 0.01^a^	0.56 ± 0.01^b^	0.51 ± 0.01^a^	0.65 ± 0.02^c^		
**Glutamic acid^f^**	16.37 ± 0.01^a^	18.56 ± 0.01^c^	19.62 ± 0.01^d^	21.15 ± 0.01^e^	18.14 ± 0.01^b^	19.61 ± 0.01^d^		
**Glycine**	5.04 ± 0.00^c^	4.71 ± 0.01^a^	6.68 ± 0.02^f^	5.16 ± 0.02^d^	5.63 ± 0.01^e^	4.90 ± 0.01^b^		
**Serine**	4.09 ± 0.01^b^	4.06 ± 0.03^b^	3.90 ± 0.01^a^	4.07 ± 0.03^b^	3.91 ± 0.01^a^	4.05 ± 0.02^b^		
**Tyrosine**	2.93 ± 0.04^e^	2.17 ± 0.03^d^	1.50 ± 0.01^a^	1.64 ± 0.01^b^	2.12 ± 0.02^c^^f^^d^	2.08 ± 0.02^c^		
**Proline**	4.42 ± 0.01^c^	5.65 ± 0.01^e^	5.39 ± 0.01^d^	3.26 ± 0.01^a^	6.39 ± 0.02^f^	3.70 ± 0.03^b^		

The data are means and standard deviations of triplicate. Column with different letters indicate statistical differences (*P* < 0.05).

a FAO/WHO/UNU. Energy and protein requirements (2007);

b Requirements for methionine + cysteine;

c Requirements for phenylalanine + tyrosine;

d Aspartic acid + asparagines;

e Cysteine + cysteine;

f Glutamic acid + glutamine. FMM–Fresh minced meat; HWD–Hot water dip.

**Table 2.** Molecular weight distribution of Tilapia fish protein hydrolysates.

**Molecular weight (Da)**	**Area (%)**					
	**Alc FMM**	**Flav FMM**	**Neut FMM**	**Alc HWD**	**Flav HWD**	**Neut HWD**
>8000	–	10.00	9.87	–	14.32	10.96
3000–8000	–	24.71	9.87	5.68	10.17	16.52
2000–3000	4.98	17.201	30.81	–	–	–
1000–2000	–	21.17	9.54	34.63	28.78	31.31
600–1000	32.47	–	20.04	–	13.17	21.57
300–600	27.87	14.72	6.91	26.9	20.43	10.69
200–300	19.54	–	5.06	–	3.28	5.80
< 200	15.11	12.83	7.87	32.79	9.85	3.38

FMM–Fresh minced meat; HWD–Hot water dip. Alc-Alcalase 2.4 L; Flav–Flavourzyme; Neut–Neutrase.

**Table 3.** Antioxidant activity of Tilapia fish protein hydrolysates.

**Sample**	**Antioxidant activity (%)**		
	**DPPH**	**ABTS**	**Fe^2+^ chelating**
**FMM**			
Alcalase	86.67 ± 1.15e	91.27 ± 0.25b	82.57 ± 0.51d
Flavourzyme	70.20 ± 1.06b	88.13 ± 0.23a	75.80 ± 0.72ab
Neutrase	82.00 ± 1.73cd	93.33 ± 0.58c	77.23 ± 0.32bc
**HWD**			
Alcalase	84.67 ± 0.58de	92.60 ± 1.30bc	78.00 ± 0.20c
Flavourzyme	64.67 ± 0.58a	93.50 ± 0.71c	75.00 ± 1.00a
Neutrase	79.67 ± 1.53c	94.23 ± 0.68c	78.32 ± 0.40c

Values are means ± standard deviation of three determinations.

Rows with different letters indicate statistical differences (*P* < 0.05).

DPPH/Chelating activity were tested at 5 mg/mL.

ABTS were tested at 66.67μg/mL.

FMM–Fresh minced meat. HWD–Hot water dip.

**Table 4.** Influence of enzyme on *In vitro* digestibility (IVD), water holding capacity (WHC), oil holding capacity (OHC), emulsifying capacity (EC), bulk density (BD) and foam capacity (FC).

**Sample**	**FMM**			**HWD**		

	**Alc.**	**Flav.**	**Neut.**	**Alc.**	**Flav.**	**Neut.**
**IVPD (%)**	92.73 ± 0.76b	88.23 ± 0.06a	92.43 ± 0.06b	93.2 ± 0.20b	89.37 ± 0.67a	92.83 ± 0.76b
**WHC (mL/g)**	2.10 ± 0.10a	2.77 ± 0.06b	2.57 ± 0.12b	1.77 ± 0.06a	2.10 ± 0.17a	2.57 ± 0.06b
**OHC (mL/g)**	2.27 ± 0.06a	3.07 ± 0.06c	2.77 ± 0.06bc	2.23 ± 0.25a	2.57 ± 0.06ab	3.13 ± 0.15c
**EC (mL/0.5g)**	22.33 ± 0.58ab	27.33 ± 0.58c	22.50 ± 0.10ab	21.40 ± 0.36a	26.40 ± 0.17c	23.20 ± 0.20b
**BD (g/mL)**	0.45 ± 0.01ab	0.35 ± 0.01a	0.53 ± 0.06b	0.53 ± 0.06b	0.34 ± 0.01a	0.46 ± 0.01ab
**FC (g/mL)**	125.50 ± 0.10de	138.50 ± 0.50b	130.20 ± 0.76c	124.50 ± 1.08e	137.50 ± 0.20a	129.60 ± 0.58d

Values are means ± standard deviation of three determinations.

Columns with different letters indicate statistical differences (*P* < 0.01).

FMMH-Fresh minced meat hydrolysate; HWDH-Hot water dip hydrolysate.

Alc–Alcalase 2.4 L; Flav–Flavourzyme; Neut–Neutrase.

**Table 5.** Characteristics used in preparation of samples in the evaluation with different proteases.

**Enzyme**	**Form**	**pH**	**T (°C)**
Alcalase 2.4 L(AU/g)*	Liquid/grain	8.0	55
Flavourzyme (500 LAPU/g)†	Powder	7.0	50
Neutrase (1.5 AU/g)	Liquid/grain	7.0	45

* AU (Anson units) is the amount of enzyme that under standard conditions digests hemoglobin at an initial rate that produces an amount of trichloroacetic acid-soluble product which gives the same color with the Filon reagent as one milliequivalent of tyrosine released per minute.

† LAPU (Leucine aminopeptidase unit) is the amount of enzyme that hydrolyze 1 μmol of leucine-*p*-nitroanilide per minute.
